# Non-Invasive Cytology Brush PCR for the Diagnosis and Causative Species Identification of American Cutaneous Leishmaniasis in Peru

**DOI:** 10.1371/journal.pone.0049738

**Published:** 2012-11-21

**Authors:** Braulio Mark Valencia, Nicolas Veland, Milena Alba, Vanessa Adaui, Jorge Arevalo, Donald E. Low, Alejandro Llanos-Cuentas, Andrea K. Boggild

**Affiliations:** 1 Instituto de Medicina Tropical "Alexander von Humboldt", Universidad Peruana Cayetano Heredia (UPCH), Lima, Peru; 2 Departamento de Ciencias Celulares y Moleculares, Facultad de Ciencias y Filosofia, Universidad Peruana Cayetano Heredia, Lima, Peru; 3 Public Health Laboratories, Public Health Ontario, Etobicoke, Ontario, Canada; 4 Department of Laboratory Medicine and Pathobiology, University of Toronto, Toronto, Ontario, Canada; 5 Hospital Nacional Cayetano Heredia, Lima, Peru; 6 Tropical Disease Unit, Division of Infectious Diseases, Toronto General Hospital, Toronto, Ontario, Canada; 7 Department of Medicine, University of Toronto, Toronto, Ontario, Canada; Queensland Institute of Medical Research, Australia

## Abstract

**Background:**

Traditional methods of detecting *Leishmania* from cutaneous lesions involve invasive diagnostic procedures, such as scrapings, which cause discomfort, require technical expertise, and carry risks of invasive procedures. We compared the performance of 2 novel, molecular-based non-invasive methods for the diagnosis of cutaneous leishmaniasis (CL).

**Methods:**

Consecutive patients presenting to the *Leishmania* Clinic at the Hospital Nacional Cayetano Heredia were enrolled. PCR was performed on filter paper lesion impressions (FPLIs), cytology brushes, and lancets for detection of *Leishmania* DNA. Smears from lesion scrapings and leishmanin skin test were also performed. Outcome measures were sensitivity and specificity. Composite reference standard was any 2 of 5 tests positive. Species identification was performed by PCR assays of positive specimens.

**Results:**

Ninety patients with 129 lesions were enrolled, 117 of which fulfilled reference criteria for a diagnosis of CL. Of these 117 lesions, 113 were positive by PCR of lancets used for lesion scrapings versus 116 by PCR of FPLIs (p = 0.930) or 116 by PCR of cytology brushes (p = 0.930). Sensitivity and specificity of PCR on lancets were 96.6% [95% CI 93.3–99.9%] and 100%, respectively. Sensitivity and specificity of FPLI PCR were 99.1% [95% CI 97.4–100%] and 100%, respectively. Sensitivity and specificity of cytology brush PCR were 99.1% [95% CI 97.4–100%] and 100%, respectively. Giemsa-stained lesion smear and leishmanin skin test had inferior sensitivities at 47.9% [95% CI 38.9–57.0%] and 82.3% [95% CI 73.9–90.7%], respectively, compared to PCR of invasive or non-invasive specimens (p<0.001).

**Conclusions:**

Cytology brush PCR constitutes a sensitive and specific alternative to traditional diagnostic assays performed on invasive specimens such as lesion scrapings. It performs comparatively to non-invasive FPLI PCR. This novel, rapid, and well-tolerated method has the potential for widespread use in the field and in pediatric populations where traditional specimen collection is difficult.

## Introduction

Accepted gold standard diagnosis of cutaneous leishmaniasis (CL) involves visualization of parasites either microscopically, or by culture, both of which traditionally involve obtaining diagnostic specimens by invasive means [1]–[3]. Invasive specimen collection can be challenging, particularly in resource-limited settings, where the disease is endemic. Scrapings and aspirates are two of the most commonly obtained clinical specimens for the diagnosis of CL, the sensitivity of which ranges from 40–75% [4]–[7] for subsequent culture to >90% for PCR [1], [7]–[12]. Invasive specimen collection techniques cause considerable discomfort, require technical expertise, carry risks of invasive procedures including bleeding and infection, and are difficult to perform in pediatric populations, in remote field settings, and are contraindicated in those with secondary bacterial or fungal infection of their ulcer due to risks of bacteremia or more complicated soft-tissue infection [1]. In addition, they pose a risk of body fluid exposure to the healthcare worker via needlestick injury and necessitate sharps biohazard procedures. The risk of sharps biohazard is a significant issue in under-resourced settings that lack the occupational health infrastructure enjoyed by health care workers in developed countries. Thus, there is a need for less-invasive, more simple and sensitive diagnostic procedures.

We have demonstrated that filter paper lesion impression (FPLI) PCR is a sensitive, well tolerated, non-invasive diagnostic approach to CL [7], [8]. We have further demonstrated that cytology brush PCR is a sensitive, non-invasive method to diagnose mucosal leishmaniasis (ML) through comparison to standard invasive methods such as biopsy histopathology and biopsy PCR [13]. However, ML and CL are vastly different clinical entities, and cytology brush PCR has yet to be validated as a diagnostic tool in CL. Although FPLI PCR is superior to invasive specimen PCR with respect to tolerability for the diagnosis of CL [7], [8], cytology brush-based diagnosis may be more amenable to easy bedside specimen collection and use in under-resourced field settings, in addition to possible applications to multiplex pathogen detection due to collection of increased cellular material.

We herein compared FPLI PCR to cytology brush PCR for the diagnosis of CL in Peruvian patients with ulcerative skin lesions, and identify important potential advantages of cytology brush PCR over FPLI PCR, including reduced risk of blood and body fluid exposure to the health care worker, and reduced risk of specimen cross-contamination. In addition, we performed species identification using PCR and RFLP on positive cytology brushes, which is essential in countries like Peru where several members of the *Leishmania (Viannia)* subgenus can cause disease and portend different prognoses [14].

## Methods

### Ethics Statement

This study was approved by the Institutional Review Boards of Hospital Nacional Cayetano Heredia (HNCH) and the University of Toronto. All patients provided written informed consent for the study procedures prior to enrolment.

### Study Site and Population

The study was conducted at the *Leishmania* Clinic of the Instituto de Medicina Tropical “Alexander von Humboldt”, Hospital Nacional Cayetano Heredia (HNCH), in Lima, Peru, between January 2011 and January 2012. Consecutive patients presenting to the *Leishmania* Clinic for the evaluation of skin lesions were approached to participate in the study, and screened for eligibility criteria. We included patients who were referred to the *Leishmania* Clinic for suspected CL; had one or more skin ulcers; and were able to give informed consent for the diagnostic procedures. We excluded patients undergoing active treatment for CL, and those with any contraindication to the diagnostic procedures.

### Lesion Sampling

#### Filter Paper Lesion Impressions

**Figure 1:**
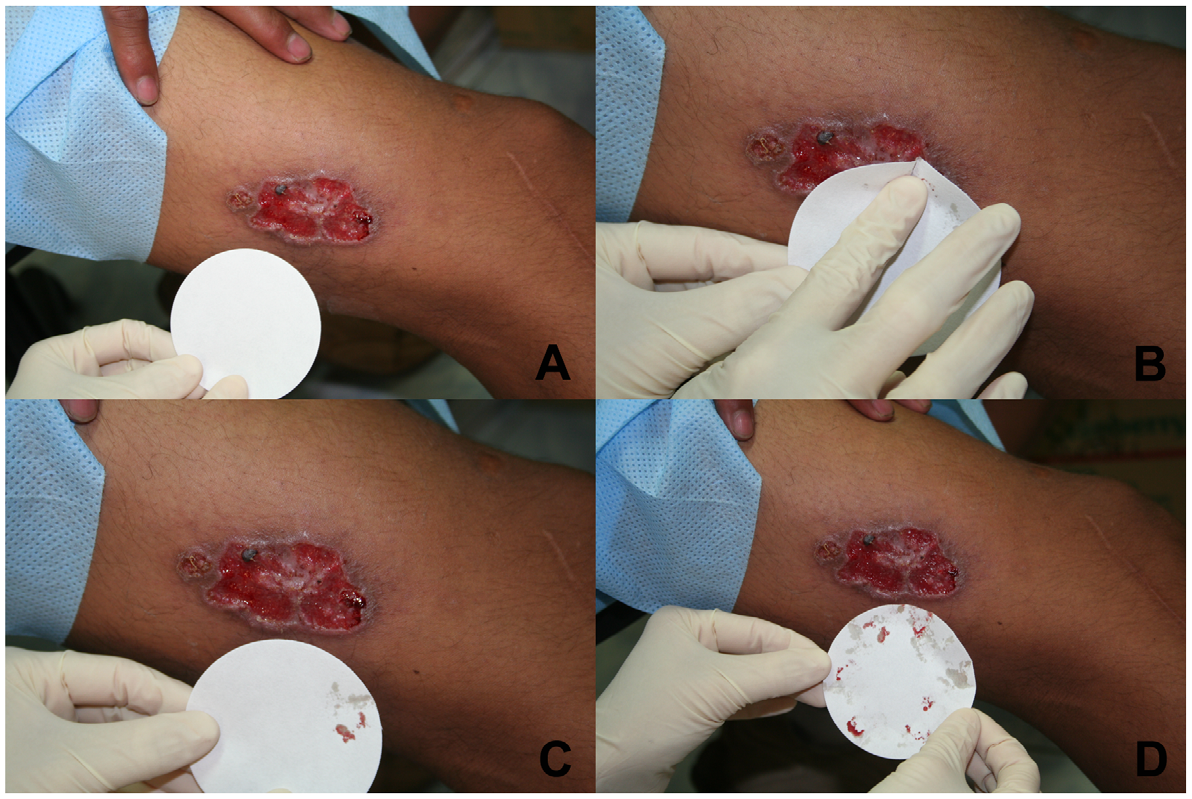
Filter paper lesion impression (FPLI) sampling method in an ulcer suspected to be cutaneous leishmaniasis. A, uninoculated filter paper; B, filter paper pressed gently onto ulcer base; C, lesion exudates wicked onto filter paper; D, filter paper with several lesion impressions and wicked exudates ready for air drying.

After removing overlying scab or crust from the ulcer with moistened gauze, single strips of sterile, Fisher brand coarse-porosity 7-cm filter paper (Fisher Scientific, Ottawa, ON) were gently pressed onto the moist ulcer base a sufficient number of times to cover the surface of the ulcer, which allows for tissue fluid to be wicked onto the filter paper (Figure 1A-D). Filter papers were then allowed to air dry, and strips were stored in 1.5-mL microcentrifuge tubes containing 700 µL 100% ethanol for qualitative PCR testing.

#### Cytology brushes

**Figure 2:**
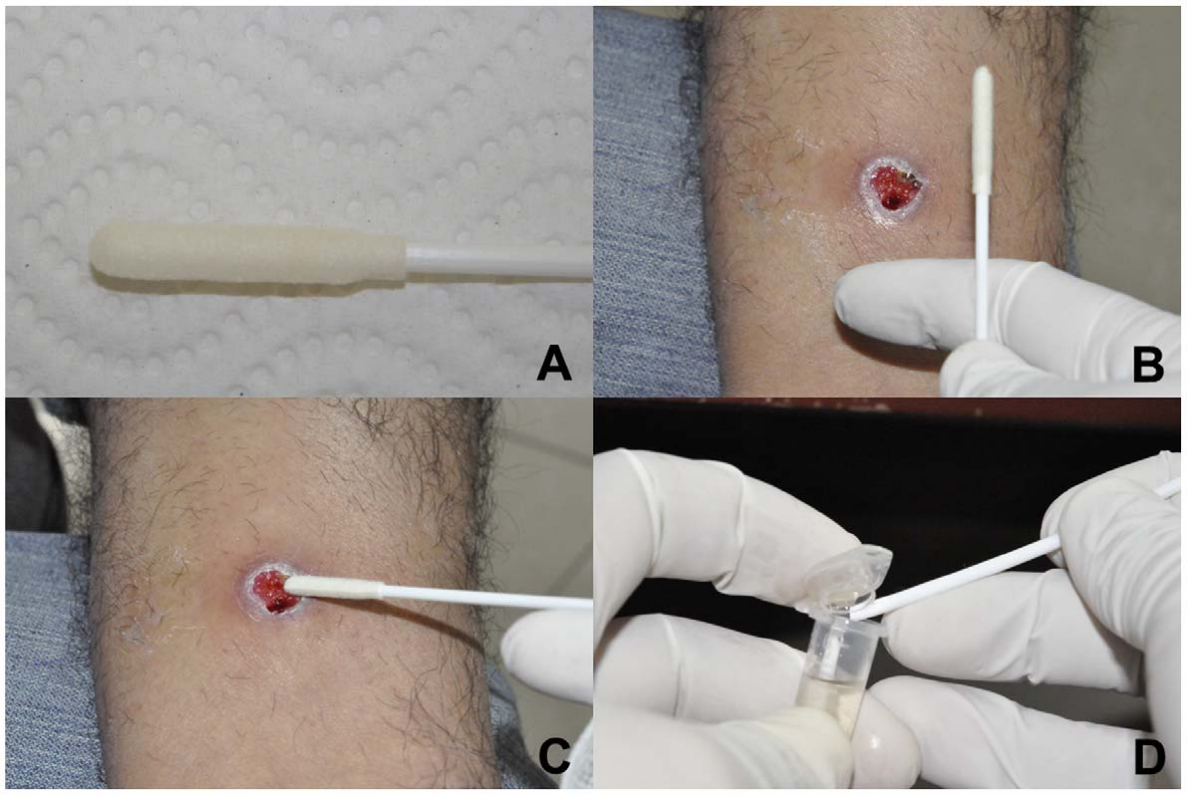
CerviSoft® cytology brush sampling method in an ulcer suspected to be cutaneous leishmaniasis. A, CerviSoft® cytology brush package and brush tip; CerviSoft® cytology brush held by health care worker in preparation for specimen collection; C, CerviSoft® cytology brush being rolled across ulcer base in order to collect lesion cellular and exudative material; D, CerviSoft® cytology brush tip broken off into a microcentrifuge tube containing 70% ethanol.

After collection of filter paper lesion impressions, sterile and duplicate CerviSoft® (Puritan Medical Products, Maine) and Histobrush® (Puritan Medical Products, Maine) cervical cytology brushes were rolled clockwise on the lesion 5 times each in sequence (Figure 2A-D). Cytology brush tips were then cut off with sterile scissors directly into 1.5-mL microcentrifuge tubes containing 700 µL 100% ethanol and stored at −20°C for qualitative PCR testing.

#### Lesion Smears

After collection of non-invasive specimens (FPLIs and brushes), lesion material was scraped from the ulcer base and border using a sterile lancet, and spread on a glass slide. Slides were air dried, fixed in methanol, and stained with Giemsa, and then examined for amastigotes under light microscopy. Lancets were stored at −20°C in 1.5-mL microcentrifuge tubes containing 700 µL 100% ethanol for qualitative PCR testing.

#### Leishmanin Skin Test

Leishmanin skin tests were applied at enrolment using 0.1 mL of in-house, sterile, heat-killed promastigote lysate in 0.005% thimerosal as described [7], [8], and read at 48 hours after administration. A positive result was indicated by ≥5 mm of erythema and induration as previously described [7], [8], [15].

#### Isolation of DNA from Cytology Brushes, Filter Papers, and Lancets

Prior to DNA extraction, samples were centrifuged at 3000 g for 5 min and ethanol was discarded. FPLIs, cytology brushes, and lancets were processed for DNA isolation using the High Pure PCR Template Preparation Kit® (Roche, Mannheim, Germany) according to manufacturer’s instructions.

#### Kinetoplast DNA (kDNA) Polymerase Chain Reaction

*Leishmania* kDNA PCR was performed using the HotStar Taq Plus DNA Polymerase kit (QIAGEN, Hilden, Germany). Final volume of the reaction mixture was 25 µL. PCR conditions were as follows: 95°C for 5 min, followed by 35 cycles of denaturation at 94°C for 30 s; primer annealing at 55°C for 30 s; extension at 72°C for 15 s, and a final extension step at 72°C for 5 min (iCycler iQ, Bio-Rad). The first primer, specific for *Leishmania (Viannia)* kDNA minicircle, had the following sequences: MP1-L (fwd) 5′ -TACTCCCCGACATGCCTCTG- 3′ and MP3-H (rev) 5′ -GAACGGGGTTTCTGTATGC- 3′, and generated a product 70 bp long [16]. Sequences of control primers, which amplify a region of the human beta-globin gene, were: HBBL (fwd) 5′-GGCAGACTTCTCCTCAGGAGTC- 3′ and HBBR (rev) 5′ -CTTAGACCTCACCCTGTGGAGC- 3′, and generated a product with a length of 197 bp. Amplicons were visualized on 3% agarose gels (Promega, Madrid, Spain) and stained with ethidium bromide.

#### Species Identification by PCR and Restriction Fragment Length Polymorphism (PCR-RFLP) of Genomic Targets

Three PCR assays targeting different sequences specific to species of the *Leishmania* (*Viannia*) subgenus including *L. (V.) braziliensis*, *L. (V.) peruviana* and *L. (V.) guyanensis*, the principal causative species in Peru, were used for species identification following initial kDNA PCR of cytology brushes. Only cytology brushes demonstrating moderate or strong bands on kDNA PCR were assayed for species identification due to an otherwise low likelihood of sufficient amplifiable DNA to confirm species. PCR assays were performed using the HotStar Taq Plus DNA Polymerase kit (QIAGEN, Hilden, Germany). Final volume of the reaction mixture was 25 µL in each case.

The first assay, targeting the mannose phosphate isomerase gene (*mpi*), consists of two separate reactions employing allele-specific reverse primers with the following sequences, which distinguish *L. (V.) peruviana* from *L. (V.) braziliensis* and *L. (V.) guyanensis*, and generates a product 312 bp long: MPI Lbp (fwd) 5′ – GCTCTTCCTGTCGGACAGCGAGC –3′ (common to all three species) and MPI Lp (rev) 5′ – GTCGGCAGCGTCACGGAGGTCC – ‘3 (specific for *L. (V.) peruviana*) or MPI Lb (rev) 5′ – GTCGGCAGCGTCACGGAGGTCG –3′ (common to *L. (V.) braziliensis* and *L. (V.) guyanensis*) [16]. *Mpi* PCR conditions were as follows: 95°C for 5 min, followed by 32 cycles of denaturation at 94°C for 30 s; primer annealing at 69°C for 30 s; extension at 72°C for 30 s, and a final extension step at 72°C for 5 min (iCycler iQ, Bio-Rad) [17].

The second assay, targeting the cysteine proteinase B (*cpb*) gene, employed primers with the following sequences, which distinguish between *L. (V.) braziliensis* and non-*L. (V.) braziliensis* species, and generated a product 1170 bp long: *Cpb* (fwd) 5′ – TGTGCTATT CGAGGAGTTCAA –3′ and *Cpb* (rev) 5′ – TTACCCTCAGGAATCACTTTGT –3′ [18], [19]. *Cpb* PCR conditions were as follows: 95°C for 5 min, followed by 45 cycles of denaturation at 94°C for 30 s; primer annealing at 60°C for 30 s; extension at 72°C for 60 s, and a final extension step at 72°C for 6 min (iCycler iQ, Bio-Rad) [18], [19].

The third assay, targeting heat shock protein 70 (*hsp70*), employed primers with the following sequences, which distinguish between *L. (V.) guyanensis* and non-*L. (V.) guyanensis* species, and generated a product 1422 bp long: *hsp70* (fwd) 5′ – GACGGTGCCTGCCTACTTCAA –3′ and *hsp70* (rev) 5′ – CCGCCCATGCTCTGGTACATC –3′ [19], [20]. *Hsp70* PCR conditions were as follows: 95°C for 5 min, followed by 45 cycles of denaturation at 94°C for 30 s; primer annealing at 60°C for 60 s; extension at 72°C for 60 s, and a final extension step at 72°C for 6 min (iCycler iQ, Bio-Rad) [19], [20].

All PCR products were visualized on 1.5% agarose gels (Promega, Madrid, Spain) and stained with ethidium bromide.

#### Restriction fragment length polymorphism analysis of cpb and hsp70 PCR products (PCR-RFLP)

Following *cpb* and *hsp70* PCR amplification as above, products were separately digested overnight at 65°C for the *cpb* assay, or 37°C for the *hsp70* assay, in a total volume of 20 µL, with 5 U of each restriction enzyme. The following enzymes were used in each reaction: *Taq*I (*cpb*) and *Hae*III (*hsp*70) (Fermentas, Burlington, Canada). Restriction fragments were then analyzed separately using 2.5% agarose gels for *cpb* or 4% agarose gels for *hsp70* (Promega, Madrid, Spain), and stained with ethidium bromide. *Mpi* PCR distinguishes *L. (V.) peruviana,* while *cpb* PCR-RFLP distinguishes *L. (V.) braziliensis*, and *hsp70* PCR-RFLP differentiates *L. (V.) guyanensis* from *L. (V.) lainsoni.*

#### Composite Reference Standard

We defined a lesion as CL when any 2 of 5 tests were positive, where tests refer to FPLI PCR; cytology brush PCR; lancet PCR; Giemsa-stained smear; or LST. These 5 tests served as the “composite reference standard” [21] against which each individual diagnostic test was compared. Assessors of smears, LST, and PCR were blind to the results of the other assays. Outcome measures were sensitivity and specificity of each assay, which were calculated in the standard manner [21].

#### Statistical Analysis

Descriptive statistics (mean, SD, median, range) were calculated for continuous variables, and differences were compared using 2-tailed t-testing. Categorical variables were quantitated by proportions, and differences between the groups were compared using Yate’s corrected Chi-square analysis. Differences in sensitivities and specificities were compared using the z-test. Statistical analyses were performed using SigmaStat 2.03 software (SPSS Inc., Chicago, IL). Level of significance was set at p<0.05.

## Results

Ninety patients with 129 ulcerative skin lesions were enrolled: 58 males and 32 females. Median age was 33 years (range 12–91 years). Median duration of lesions was 3 months (range 2 weeks –12 months). Twenty-nine patients (32%) presented with multiple lesions, with a median number of lesions per patient of 1 (range 1–10). Two participants (2.2%) had evidence of intercurrent mucosal and cutaneous involvement. Lesions were primarily located on the lower extremity (38%), upper extremity (34.9%), or face (11.6%). Ulcers had median dimensions of 2.2-cm (range 0.4–10.0-cm) by 1.8-cm (range 0.4–9.0-cm).

Using the composite standard (at least 2/5 tests positive), 117 lesions (90.7%) fulfilled criteria for a diagnosis of CL. 118 lesions (91.5%) were positive by at least one test, 114 (88.4%) were positive by 3 or more tests, 103 (79.8%) were positive by 4 or more tests, and 48 (37.2%) were positive by all 5 tests. When the individual patient was used as the unit of analysis, sensitivities and specificities of individual assays did not change appreciably from the per-lesion analysis, and statistically significant differences remained significant.

### Smear and LST

Of the 117 lesions that were positive by at least 2 of 5 diagnostic tests, 56 were smear positive. The sensitivity and specificity of smear was 47.9% [95% CI 38.9–57.0%] and 100%, respectively [Table 1]. 65 patients with 97 enrolled lesions had positive LSTs, yielding a sensitivity and specificity of 82.3% (95% CI 73.9–90.7%) and 100%, respectively [Table 1].

**Table 1:** Analysis of 5 Diagnostic Tests used in the Evaluation of 129 Lesions Suspected to be Cutaneous Leishmaniasis in 90 Peruvian patients.

Assay	Number Positive	Number Negative	Sensitivity (%)	Specificity (%)	PPV (%)	NPV (%)
LST*	65	25	82.3	100.0	100.0	44.0
Smear	56	73	47.9	100.0	100.0	16.4
kDNA PCR of Lesion Scrapings	113	16	96.6	100.0	100.0	75.0
kDNA PCR of FPLIs	116	13	99.1	100.0	100.0	92.3
kDNA PCR of CerviSoft® cytology brushes	117	12	99.1	91.7	99.1	91.7
kDNA PCR of Histobrush® cytology brushes	115	14	98.3	100.0	100.0	85.7

* per patient analysis.

Abbreviations: FPLI, filter paper lesion impression; LST, leishmanin skin test; NPV, negative predictive value; PPV, positive predictive value.

### PCR of Scrapings

113 lesions of 117 fulfilling reference criteria for CL were positive by kDNA PCR of invasively obtained lesion scrapings, yielding a sensitivity of 96.6% (95% CI 93.3–99.9%) [Table 1]. Compared to the composite standard, specificity of PCR scrapings was 100% [Table 1]. PCR of invasive scrapings was more sensitive than LST or smear (p<0.001).

### PCR of Filter Paper Lesion Impressions

116 lesions were positive by kDNA FPLI PCR, yielding a sensitivity and specificity of 99.1% (95% CI 97.4–100%) and 100%, respectively [Table 1]. PCR of FPLIs was more sensitive than LST or smear (p<0.001). PCR of FPLIs was equally sensitive as PCR of scrapings (p = 0.34) and cytology brush PCR (p = 0.958).

### PCR of Cytology Brush Specimens

117 lesions were positive by kDNA PCR of CerviSoft cytology brushes, yielding a sensitivity and specificity of 99.1% (95% CI 97.4–100%) and 91.7% (95% CI 76.1–100%), respectively [Table 1]. 115 lesions were positive by PCR of Histobrush cytology brushes, yielding a sensitivity and specificity of 98.3% (95% CI 96.0–100%) and 100%. PCR of cytology brushes was more sensitive than LST or smear (p<0.001).

### Species Identification

Of 118 CL lesions with at least one kDNA PCR-positive cytology brush, sufficient amplifiable DNA (ie, moderate to strong banding on kDNA assay) for definitive species identification by subsequent PCR and RFLP was present in 109 (92.4%). Of 91 kDNA-positive cytology brushes with definitive species results (83.5%), species identification was as follows: *L. (V.) braziliensis*, 21 lesions; *L. (V.) peruviana*, 32 lesions; *L. (V.) guyanensis*, 32 lesions; and *L. (V.) lainsoni*, 6 lesions [Table 2]. In 7 sets of cytology brushes, discrepant species identification results occurred such that one brush yielded a “not identifiable” result and the other a definitive species identification (6 Cervisoft® brushes, and 1 Histobrush®). Thus, the sensitivities of Cervisoft® and Histobrush® brushes for definitive species identification were 73.5% [95% CI 65.5–81.5%] and 76.9% [95% CI 69.3–84.5%], respectively, with 100% specificity in both.

**Table 2:** Species identification of 91 out of 118 kDNA PCR-positive lesions subsequently tested with PCR targeting the mannose phosphate isomerase, cysteine proteinase B and heat shock protein 70 genes and subsequent RFLP.

*Leishmania* Species	Number (% of those tested)
*L. (V.) braziliensis*	21 (19.3%)
*L. (V.) guyanensis*	32 (29.4%)
*L. (V.) lainsoni*	6 (5.5%)
*L. (V.) peruviana*	32 (29.45)
Not identifiable	18 (16.5%)
Not tested*	9

* Only specimens with sufficient amplifiable DNA from the kDNA PCR assay were selected for species identification PCR assays. These 9 specimens had a positive cytology brush kDNA PCR but insufficient genomic DNA concentration for species identification based on weak banding pattern.

## Discussion

We have demonstrated in a clinical evaluation of ulcerative lesions suspected to be CL in Peru that commercial grade cervical cytology brushes offer comparable diagnostic sensitivity and specificity as FPLI and invasive specimen PCR, and outperform conventional non-molecular diagnostic tests such as LST and smear. While the performance characteristics of PCR on each specimen were comparable, cytology brush PCR has the advantage over scraping PCR of being completely non-invasive. Thus, it requires little technical expertise, carries none of the risks associated with incising skin, and can be easily performed in pediatric patients. Furthermore, there are no associated sharps biohazard risks to the healthcare worker collecting the diagnostic specimen, which cannot be said for traditional invasive diagnostic specimens such as scrapings, aspirates, and biopsies. Reducing biohazard risks to healthcare workers is desirable as CL endemic areas are typically under-resourced and lacking in occupational health infrastructure, protocols, and protections. Thus, eliminating needle stick and sharps injuries to local healthcare workers should not be overlooked as a goal when developing novel diagnostics.

Cytology brush PCR had similar performance characteristics as FPLI PCR in both per-lesion and per-patient analyses. FPLI PCR is a novel, non-invasive molecular diagnostic method that has been pioneered and validated by our group [7], [8], [22]. While this type of specimen is also collected non-invasively, and is therefore superior to traditional invasive scrapings and aspirates, actual collection and post-collection processing of the FPLIs requires direct handling of the specimen by the technician or healthcare worker, and lesion contact. Although this procedure does not create a blood and body fluid risk to the diagnostician if gloves are worn and remain intact, it can facilitate cross-contamination of specimens if strict inter-specimen decontamination procedures for gloves, processing equipment, and the local environment are not adhered to [7]. Requiring such strict decontamination procedures is time consuming and vulnerable to error. Thus, the ideal collection point of a non-invasive diagnostic specimen would not come in contact with healthcare worker gloves or the local environment after collection. Cytology brushes fulfill this requirement as only the distal tip of the brush contacts the lesion, and then is promptly severed into ethanol by breakage of the wand or cutting with scissors. Cytology brush tips containing cellular material and tissue fluid from lesions do not need to air dry before further processing and can be collected and prepared at point-of-care.

Another desirable characteristic of novel, non-invasive diagnostic specimens is their ease of transport from the field (rural endemic areas) to diagnostic reference centers, which are typically located in non-endemic urban centers such as Lima. Although not specifically addressed by this diagnostic evaluation, cytology brush tips (and FPLIs) could be theoretically stored for more prolonged periods of time and easier to transport in 1.5-mL microcentrifuge tubes at room temperature than would glass slides for Giemsa-stained microscopy, or material for culture. At present, many remote endemic areas offer only lesion smear, if any diagnostic work-up is performed at all. Future validation of these non-invasive specimen collection methods in remote endemic areas is warranted, since many health centers in endemic areas of Peru lack basic facilities such as electricity and refrigeration.

We have demonstrated that cytology brush PCR is adequate for diagnosis of CL and have validated its use for causative species identification, a necessity in countries like Peru where multiple *Leishmania* species co-exist. The diagnostic kDNA PCR employed herein targets a multicopy minicircle conserved region of kDNA common to *Leishmania* (*Viannia*) species [16]. There are up to 10,000 copies of this target per amastigote. Conversely, our species identification algorithm relies on PCR-based assays whose targets are nuclear DNA with only 2–6 copies per amastigote, and is therefore much less sensitive than the diagnostic kDNA PCR. In order to improve the sensitivity of the PCR assays for species identification, collection of additional cellular material (containing additional amastigotes) is desirable. Although not directly tested, the sensitivity of cytology brush PCR for species identification is likely superior to that of FPLI PCR given the higher concentration of cellular material in the specimen rather than just tissue fluid. This is corroborated by our findings herein and previously: while FPLI PCR and RFLP was able to identify causative species in 54/108 (50%) kDNA PCR-positive filter papers [7], [8], cytology brush PCR-RFLP was able to identify causative species in 91 (77%) lesions enrolled herein. Use of this PCR and RFLP algorithm on non-invasive specimens [22] constitutes a major advance in our approach to species identification, which has historically required a multi-step process using cultured promastigotes and labour-intensive isoenzyme analysis [18].

In summary, we have demonstrated that cytology brush PCR using CerviSoft® and Histobrush® cervical cytology brushes for the diagnosis of CL is simple, rapid, potentially portable, and extremely well tolerated by patients. In addition, cytology brush PCR offers economic advantages over invasive-specimen PCR by obviating the need for anesthesia, the bedside consumables needed to control bleeding, and sharps biohazard containers. At just 30–50 cents (US) per cytology brush, this novel diagnostic specimen is practical and comparatively affordable. In reference centers, cytology brush PCR should be viewed as superior to traditional diagnostic tests such as smear and LST, and at least comparable to molecular testing of invasive specimens such as scrapings. Further validation of this technique as it applies to field settings or in non-ulcerative lesions is required.
